# On Cup Anemometer Rotor Aerodynamics

**DOI:** 10.3390/s120506198

**Published:** 2012-05-10

**Authors:** Santiago Pindado, Javier Pérez, Sergio Avila-Sanchez

**Affiliations:** IDR/UPM, ETSI Aeronáuticos, Universidad Politécnica de Madrid, Pza. del Cardenal Cisneros 3, Madrid 28040, Spain; E-Mails: javier.perez@upm.es (J.P.); s.avila@upm.es (S.A.-S.)

**Keywords:** anemometer calibration, cup anemometer, cup aerodynamics, rotor aerodynamics

## Abstract

The influence of anemometer rotor shape parameters, such as the cups' front area or their center rotation radius on the anemometer's performance was analyzed. This analysis was based on calibrations performed on two different anemometers (one based on magnet system output signal, and the other one based on an opto-electronic system output signal), tested with 21 different rotors. The results were compared to the ones resulting from classical analytical models. The results clearly showed a linear dependency of both calibration constants, the slope and the offset, on the cups' center rotation radius, the influence of the front area of the cups also being observed. The analytical model of Kondo *et al.* was proved to be accurate if it is based on precise data related to the aerodynamic behavior of a rotor's cup.

## 1. Introduction

Rotation anemometers, such as cup and propeller anemometers, are the most commonly used instruments for wind speed measurements. Thanks to their linearity and accuracy they are optimal for a large number of applications in the wind energy sector, from routine observations to field measurements. Cup anemometers have been widely studied since the first half of the twentieth century, with the early works devoted to studying the optimal number of cups and arm length [1,2]. Additionally, the cups' rotor aerodynamics and its behavior, especially in turbulent wind, was the subject of great interest throughout the twentieth century. Wyngaard [3] used a 2-cup model to illustrate the “overspeeding” effect of the anemometer, that is, the cup anemometer responds quicker to wind accelerations than to wind decelerations. Other interesting models were developed by Ramachandran [4] and Kondo *et al.* [5], to analyze the cups' rotor dynamics as a function of the aerodynamic drag of the cups. Both authors referred to the research by Brevoort and Joyner [6,7] for this cup aerodynamic drag.

In a previous study at the IDR/UPM Institute [8] large series of calibrations were analyzed, the calibration coefficients of the anemometers' transfer function being studied as a function of the anemometers' shape. The transfer function of an anemometer is represented by the following expression:

(1) V=A⋅f+B

where *V* is the wind speed, *f* is the anemometer's rotation frequency output, and A (slope) and B (offset) are the calibration coefficients. This linear equation, which correlates the wind speed and the anemometer's output frequency [9], has to be defined by means of a calibration process. The aforementioned transfer function can be rewritten in terms of the anemometers' rotation frequency, *f_r_*, instead of the output frequency *f*, as:

(2) $$V={\text{A}}_{r}\cdot f_{r}+\text{B}$$

where A*_r_* is the result of multiplying the calibration constant A by the number of pulses per revolution given by the anemometer, *N_p_*. The number of pulses is different depending on the anemometer's inner system for translating the rotation into electric pulses. Magnets-based systems give from 1 to 3 pulses per revolution, whereas opto-electronics-based systems normally give higher pulse rates per revolution, from 6 to 44 [8].

**Figure 1.**
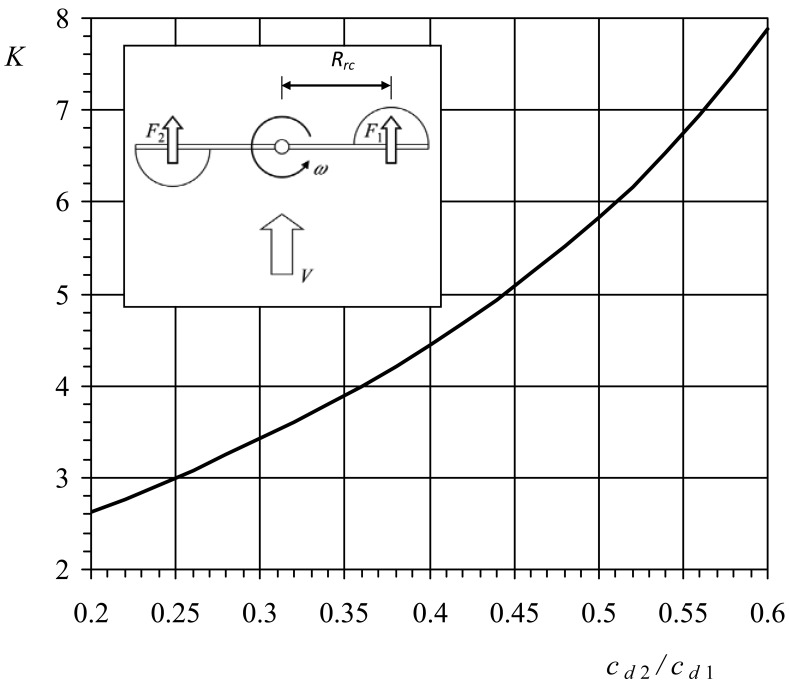
2-cup anemometer model: anemometer factor (see Section 1.1), *K*, as a function of the ratio between the aerodynamic drag coefficient of the cups at 0° wind angle, *c_d_*_1_, and at 180° wind angle, *c_d_*_2_.

This research, performed on more than 20 models of commercial cup anemometers, showed a linear correlation between the coefficients A*_r_* and the cups' center rotation radius (defined in Figure 1), *R_rc_*, A*_r_* = 0.012*R_rc_* + 0.546 (*R_rc_* expressed in mm). However, this linear fitting changed to A*_r_* = 0.019*R_rc_* + 0.196, with a better regression coefficient (*R*^2^ = 0.753 instead of *R*^2^ = 0.485, in the previous fitting) leaving aside some anemometers, those that have very different shape from the others, when calculating the linear fitting. Finally, a brief calculation using the 2-cup analytical method showed a very close result to the mentioned fitting, A*_r_* = 20.7*R_rc_* (*R_rc_* expressed in meters). On the other hand, based on the analysis, B calibration coefficients of the studied anemometer models did not seem to correlate to the cups' center rotation radius, *R_rc_*, nor to the cups' front area, *S_c_*.

### 1.1. The 2-Cup Model

**Figure 2.**
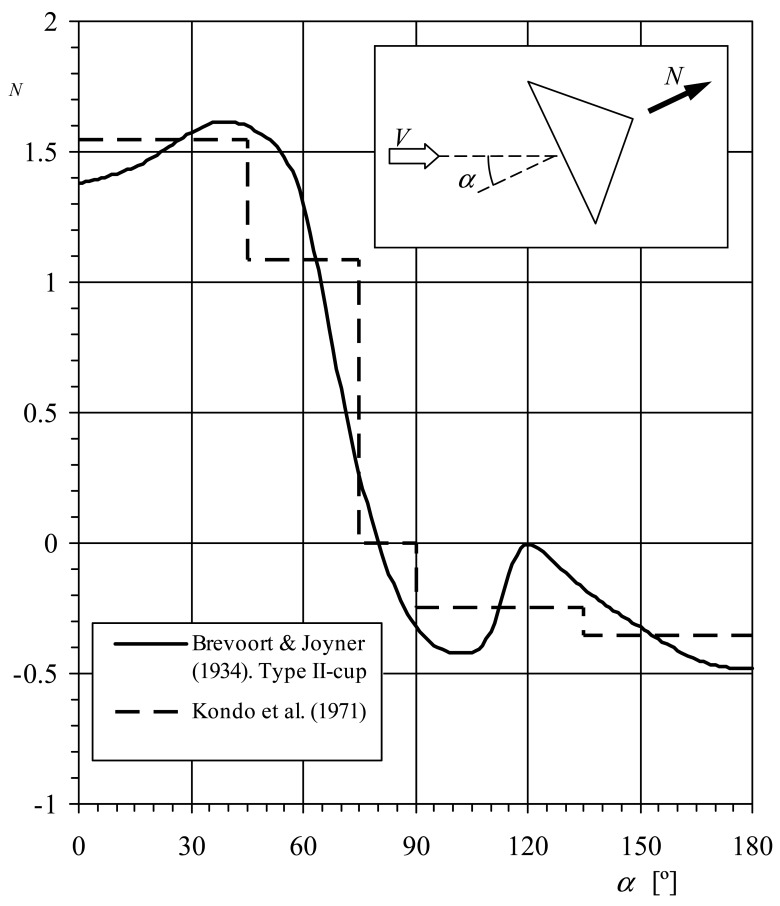
Normal aerodynamic force coefficient, *c_N_*, of the Brevoort and Joyner Type-II cup plotted as a function of the wind direction with respect to the cup, *α*.

This simple model was used in the past to study the aerodynamics of the cup anemometers' rotor (see a sketch of the model in the Figure 1). This model is based on the perfect rotor equilibrium assumption, that is, the aerodynamic torque is neglected (see [1,3]). The response of a cup anemometer can be derived from the following expression [10]:

(3) $$I\frac{d\omega }{dt}=Q_{A}+Q_{\text{f}}$$

where *I* is the moment of inertia, *Q_A_* is the aerodynamic torque, and *Q*_f_ is the frictional torque. The frictional torque, *Q*_f_, can be neglected as it is usually (for wind speeds larger than 1 m·s^−1^) very small in comparison to the aerodynamic torque, *Q_A_* [11,12]. So finally, the response can be obtained if the aerodynamic torque is assumed to be equal to zero, that is, *F*_1_ = *F*_2_, where *F*_1_ and *F*_2_ are the aerodynamic forces on the cups (see Figure 1). This condition can be expressed as:

(4) $$\frac{1}{2}\rho {\left(V-\omega R_{rc}\right)}^{2}c_{d1}S_{c}=\frac{1}{2}\rho {\left(V+\omega R_{rc}\right)}^{2}c_{d2}S_{c}$$

where *V* is the wind speed, *ω* is the rotor's angular velocity, *R_rc_* is the cups' center rotation radius, *S_c_* is the front area of the cups, and *c_d_*_1_ and *c_d_*_2_ are the drag coefficients of the cups, respectively at 0° and 180° regarding the wind direction (the wind direction angle with respect to the cup is indicated in Figure 2). This equation can be simplified to obtain the transfer function of the anemometer:

(5) $$V=2\pi \left(\frac{1+\sqrt{c_{d2}/c_{d1}}}{1-\sqrt{c_{d2}/c_{d1}}}\right)R_{rc}f_{r}={\text{A}}_{r}f_{r}$$

The ratio between the wind speed, *V*, and the rotation speed of the cups' center, *ωR_rc_*, is called the anemometer factor, *K*:

(6) $$K=\frac{V}{\omega R_{rc}}=\frac{{\text{A}}_{r}}{2\pi R_{rc}}=\left(\frac{1+\sqrt{c_{d2}/c_{d1}}}{1-\sqrt{c_{d2}/c_{d1}}}\right)$$

From an early study by Patterson, this factor was found to be between 2.5 and 3.5 [9]. Based on the data from [8], most common commercial anemometers have factors between 2.97 and 3.54 (calculated leaving aside the offset of the transfer function). In Figure 1, the *K* factor estimated with this 2-cup model is shown as a function of the ratio *c_d_*_2_/*c_d_*_1_.

### 1.2. The Ramachandran Model

Ramachandran [4] derived the rotor's behavior from the normal aerodynamic force coefficient of a single cup, *c_N_*, (see in Figure 2, a sketch regarding the aerodynamic normal force on a cup in relation to the wind speed direction). Taking into account the three cups of a rotor, the Equation (3) can be rewritten as:

(7) $$I\frac{d\omega }{dt}=\frac{1}{2}\rho S_{c}R_{rc}V_{r}^{2}(\theta )c_{N}\left(\alpha \left(\theta \right)\right)+\frac{1}{2}\rho S_{c}R_{rc}V_{r}^{2}(\theta +120{}^{\circ})c_{N}\left(\alpha (\theta +120{}^{\circ})\right)+\frac{1}{2}\rho S_{c}R_{rc}V_{r}^{2}((\theta +240{}^{\circ})c_{N}\left(\alpha (\theta +240{}^{\circ})\right)$$

where *V_r_* is the relative wind speed to the cups, *α* is the wind direction with respect to the cups, and *θ* is the angle of the rotor with respect to a reference line (see in Figure 4, a more detailed sketch with regard to these variables). This approximation leaves aside the aerodynamic moment of the cups, as its contribution to the rotor's torque is, in principle, less important (this effect was checked in the present research). The relative wind speed, *V_r_*, to the cup at *θ* rotor angle with respect to the reference line can be expressed as:

(8) $$V_{r}\left(\theta \right)=\sqrt{V^{2}+{\left(\omega R_{rc}\right)}^{2}-2V\omega R_{rc}\cos\left(\theta \right)}$$

whereas the wind direction with respect to the cup, *α*, can be derived from these two equations:

(9) $$V_{r}\left(\theta \right)\sin\left(\alpha \right)=V\sin\left(\theta \right)$$

(10) $$V_{r}\left(\theta \right)\sin\left(\alpha -\theta \right)=\omega R_{rc}\sin\left(\theta \right)$$

to the following expression:

(11) $$\tan\left(\alpha \right)=\frac{K\sin\left(\theta \right)}{K\cos\left(\theta \right)-1}$$

Ramachandran made two important assumptions in his calculations:

(12) $$V_{r}\left(\theta \right)\approx V-\omega R_{rc}\cos\left(\theta \right)$$

and:

(13) α≈θ

**Figure 3.**
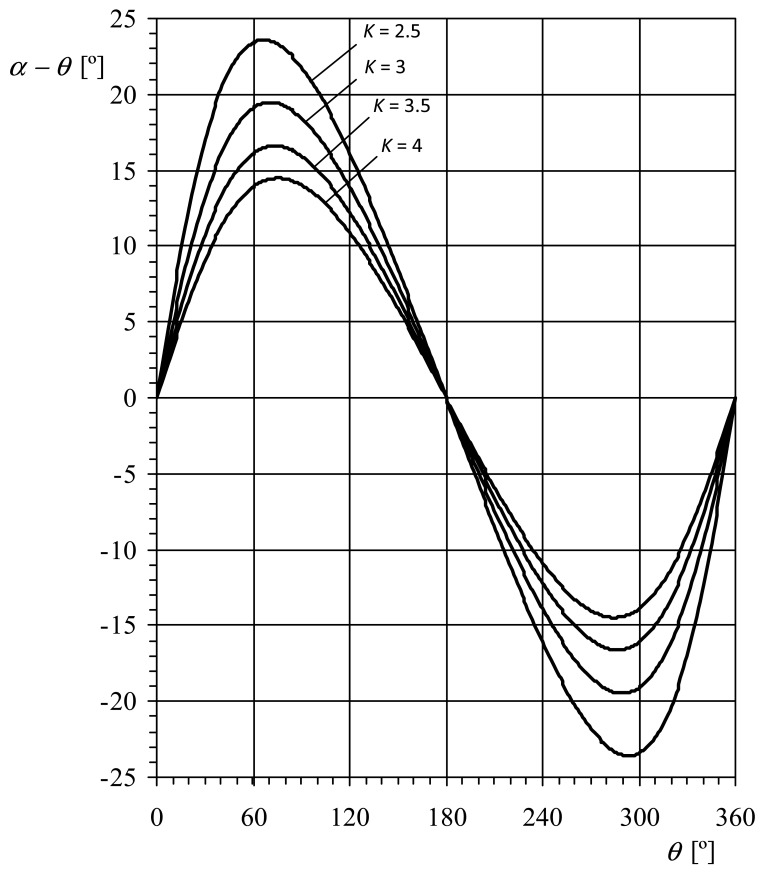
Difference between the local wind angle with respect to the cup (commonly known as angle of attack), *α*, and the anemometer rotor's rotation angle, *θ*, as a function of the latter, for different anemometer factors, *K*. See also the sketch included in Figure 4.

Taking into account the aforementioned ratios between the wind speed and the rotation speed (that is, the anemometer factor, *K*), it can be observed that Equation (12) does not deviate excessively from the exact Equation (8) (up to 8.3%, 5.7% and 4.2%, respectively for factors *K* = 2.5, 3 and 3.5). However, the second assumption, indicated with the Equation (13), involves more important deviations (up to 23.6°, 19.5° and 16.6° for the mentioned values of *K*, see Figure 3).

The anemometer's behavior in steady state can be obtained from Equation (7) by making its average value equal to zero in one turn, that is:

(14) $$V=4\pi R_{rc}\frac{b_{m}}{a_{m}}f_{r}={\text{A}}_{r}f_{r}$$

where the ratio *b_m_*/*a_m_* can be simplified as:

(15) $$\frac{b_{m}}{a_{m}}=\frac{\int_{0}^{2\pi }c_{N}\left(\alpha \right)\cos\left(\alpha \right)d\alpha }{\int_{0}^{2\pi }c_{N}\left(\alpha \right)d\alpha }$$

As mentioned, in order to calculate the last expression Ramachandran suggested the use of Brevoort and Joyner results [6,7]. These authors measured the normal aerodynamic force coefficient of anemometer cups, *c_N_*, as a function of the wind angle, *α*, for five cup shapes (named in the referred reports: Type I-hemispherical, without bead, 4.03 in diameter-, Type II-conical, without bead, 4.56 in diameter-, Type III-hemispherical, with bead, 2.03 in diameter-, Type IV-conical, with bead, 4.70 in diameter-, and Type V-hemispherical, without bead, 6.00 in diameter-). In Figure 2, the normal force coefficient, *c_N_*, related to the Type II cup, is shown as a function of the wind direction with respect to the cup, *α*. Solving Equations (14) and (15) with this normal force coefficient distribution, the anemometer's factor becomes *K* = 2.64, and the calibration coefficient A*_r_* = 16.6*R_rc_* (*R_rc_* expressed in meters). This value of the calibration coefficient is lower than the one resulting from the previous research (A*_r_* = 19·*R_rc_*) [8].

**Figure 4.**
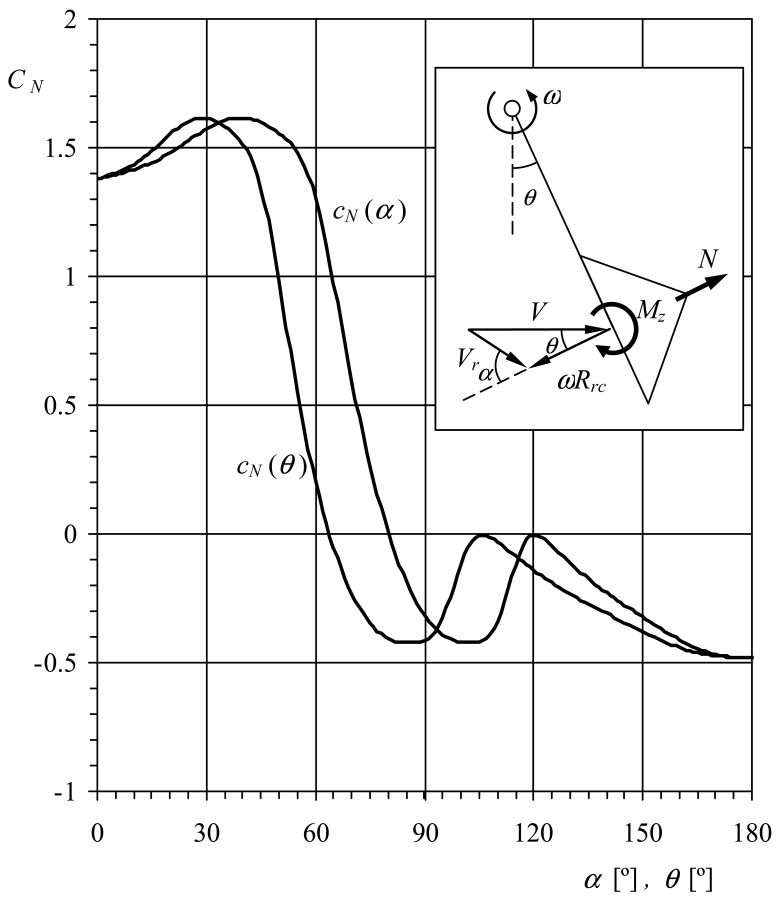
Normal aerodynamic force coefficient, *c_N_*, of the Brevoort and Joyner Type-II cup plotted as a function of the wind direction with respect to the cup, *α*, and the rotor's rotation angle, *θ*. Calculated for anemometer factor *K* = 3.5.

Kondo *et al.* [5] proposed a solution to the problem considering the anemometer's rotor position, *θ*, different from the local wind angle with respect to the cup, *α*. See in Figure 4, the normal aerodynamic force coefficient of the Brevoort and Joyner Type-II cup plotted as a function of both angles, *α* and *θ*, together with a sketch regarding the geometric relations between *V, ωR_rc_, α*, and *θ*. Again, averaging Equation (7) and making the result equal to zero, it is possible to obtain the following equation:

(16) $$0=\left(V^{2}+{\left(\omega R_{rc}\right)}^{2}\right)\int_{0}^{2\pi }c_{N}\left(\alpha \left(\theta \right)\right)d\theta -2V\omega R_{rc}\int_{0}^{2\pi }c_{N}\left(\alpha \left(\theta \right)\right)\cos\left(\theta \right)d\theta$$

that leads to the solution of the anemometer's steady state:

(17) $$K=\frac{V}{\omega R_{rc}}=\frac{b}{a}+\sqrt{{\left(\frac{b}{a}\right)}^{2}-1}$$

where:

(18) $$\frac{b}{a}=\frac{\int_{0}^{2\pi }c_{N}\left(\alpha \left(\theta \right)\right)\cos\left(\theta \right)d\theta }{\int_{0}^{2\pi }c_{N}\left(\alpha \left(\theta \right)\right)d\theta }$$

Kondo *et al.* simplified the calculations by using a stepwise line for the coefficient *c_N_*(*α*) (see Figure 2). However, it is possible to express this coefficient as a function of *θ* using the Equation (11) together with a reasonable value of the factor *K*, and then solve Equations (17) and (18) in order to obtain a more accurate value of this factor. Using this method it is possible to get the solution in a few iterations. In the present case *K* = 3.5, and the calibration coefficient A*_r_* = 22.02*R_rc_* (*R_rc_* expressed in meters).

The aim of the present work is to analyze the correlation between the cups' rotor dynamics in steady state (that is, the anemometer's transfer function) and the rotor's shape (more specifically, the cups center rotation radius, *R_rc_*, and the cups' front area, *S_c_*), by means of a specific testing research. Other effects such as the cups' aerodynamic drag coefficient measured both on one isolated cup and on one cup surrounded by the other two inside the rotor, are also taken into account. The testing campaign was divided into two parts, the first one included calibrations performed on two different anemometers with 21 different rotors (varying both the cups' size and the rotation radius), whereas the second one included the aerodynamic forces measurements on a cup, isolated and in a rotor (that is, surrounded by the other two). In order to have a better understanding of the anemometers' rotor dynamics, the results were compared to the aforementioned classic analytical models.

## 2. Testing Configuration and Cases Studied

**Figure 5.**
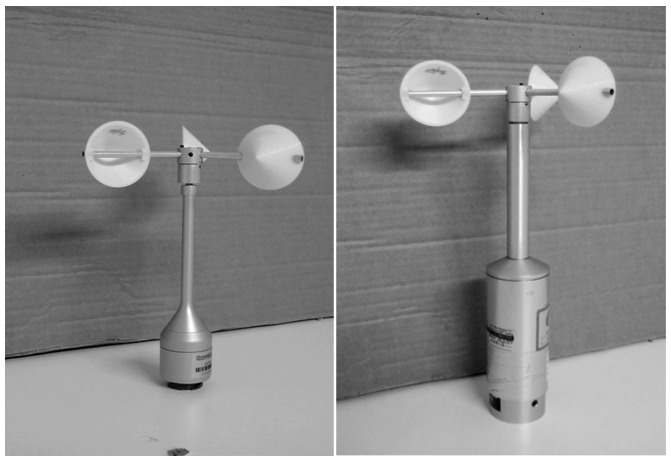
Ornytion 107A (**left**) and Climatronics 100075 (**right**) anemometers, both with 50/60 rotor (see in Table 1 the characteristics of each rotor tested).

**Figure 6.**
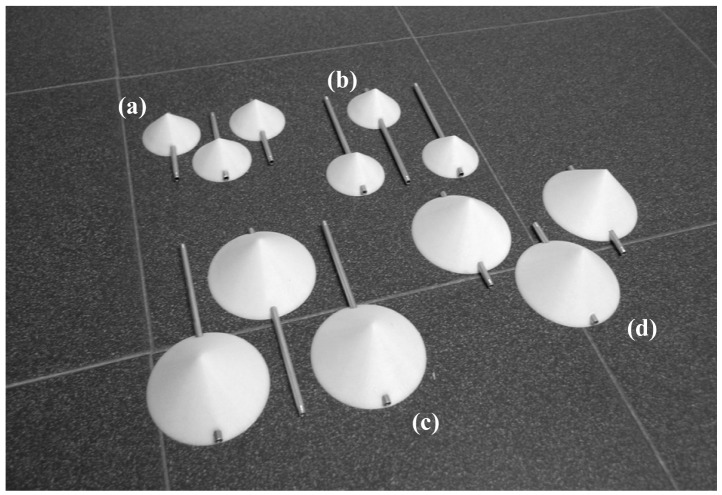
Cups sets correspondent to the 50/60 (**a**), 50/100 (**b**), 80/120 (**c**) and 80/60 (**d**) rotors (see in Table 1 the characteristics of each rotor tested).

Two anemometers, Climatronics 100075 (also known as F460 model, by Climatronics Corp.: Bohemia, New York, USA), and Ornytion 107A (Ornytion: Bergondo, A Coruña, Spain) were used in the testing campaign (see Figure 5). These anemometers will be referred hereinafter in the text as Cl-100075 and Ory-107 respectively. As said, 21 different rotors were tested on both anemometers (see Figure 6), varying the cups' front section, *S_c_*, and the cups' center rotation radius, *R_rc_*, from one another (see Table1). All cups tested were conical (90° cone-angle). The cups were made in a 3D printer of ABS plastic, and the arm of each cup was made of 5 mm diameter aluminum tube. Each set of three cups were attached to the Cl-100075 anemometer rotor's head. In order to use this head with the Ory-107 anemometer, a special piece had to be designed and manufactured as an interface to the Ory-107 shaft. These anemometers have different electronic systems, the Cl-100075 is equipped with an opto-electronic system that gives 30 squared pulses per rotation, while the Ory-107 has a magnets-based system that gives two harmonic pulses per rotation.

The calibrations were carried out at the IDR/UPM Institute, in the S4 wind tunnel. This facility is an open-circuit wind tunnel with a closed test section measuring 0.9 by 0.9 m. It is served by four 7.5 kW fans with a flow uniformity under 0.2% in the testing area. More details concerning the facility and the calibration process are included in references [8,13]. The calibrations analyzed in the present paper were performed following the MEASNET [14,15] recommendations (over 13 points and from 4 to 16 m·s^−1^ wind speed).

**Figure 7.**
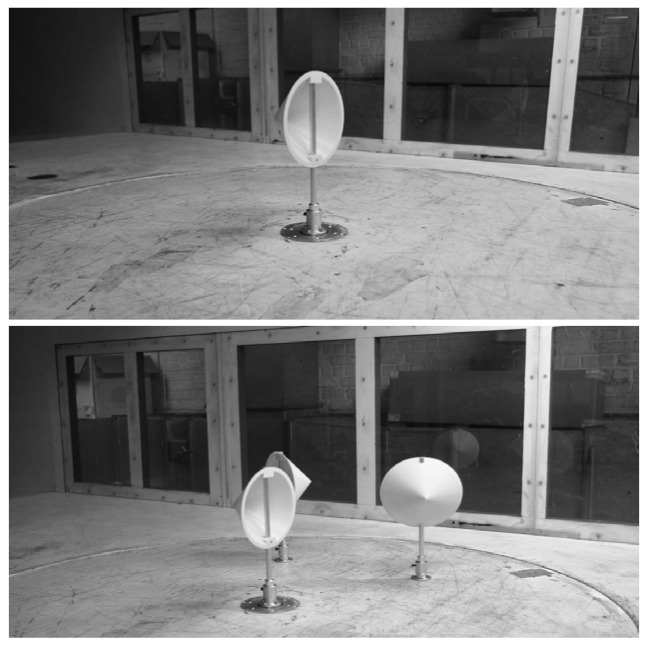
Both cup configurations measured in the wind tunnel of the Vrije Universiteit Brussel: isolated cup (**top**), and cup inside a 3-cup rotor (**bottom**).

The aerodynamic forces on the cups were measured in the wind tunnel of the Department of Mechanical Engineering of the Vrije Universiteit Brussel (Belgium). This facility is also an open-circuit wind tunnel with a 2 by 1 m closed test section. The facility is served by a 55 kW centrifugal fan. The testing section is equipped with a 6-component balance made by TEM Engineering Limited (Sussex, UK). Three larger-scale cups (0.2 m diameter) were manufactured to measure the aerodynamic forces. These cups were also made in a 3D printer of ABS plastic, and they are an exact scale-replica of the rotors' ones. As stated in the introduction, two different tests were carried out. In the first one, the forces on an isolated cup were measured by varying the wind direction, whereas in the second one the cup was surrounded by the other two in order to better simulate the anemometer's rotor (see Figure 7). These tests were carried out in smooth flow (with low turbulence, 1–1.5%), with around 15 m·s^−1^ wind speed. 12,000 samples were taken at 50 Hz in each measurement. The forces were made non dimensional with the dynamic pressure directly measured by a BnC-Lambrecht 630a (Goettingen, Germany) pitot tube located at the ceiling of the testing chamber, upstream to the point where the models are allocated, and connected to a SETRA Model 239 (Boxborough, MA, USA) differential pressure sensor.

## 3. Results and Discussion

**Figure 8.**
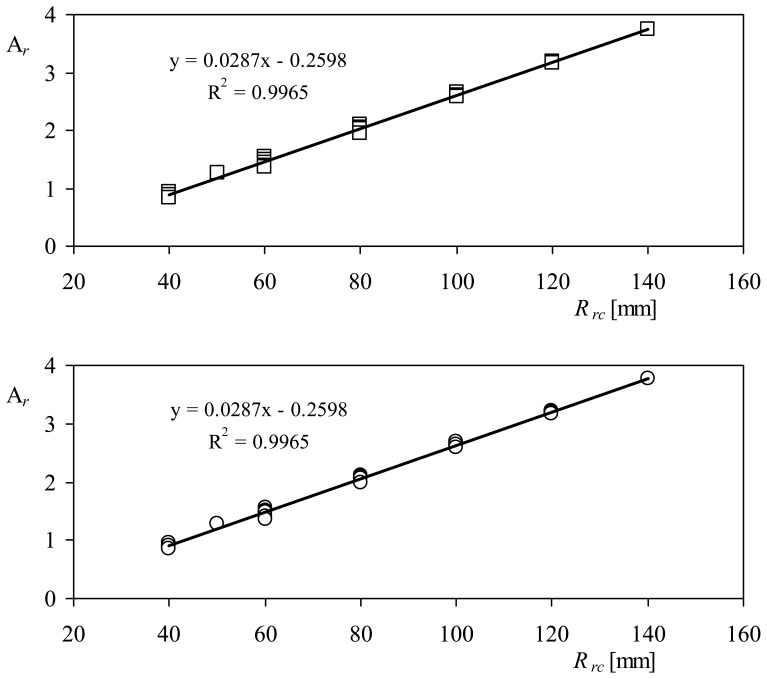
Calibration coefficients, A*_r_*, as a function of the cups' center rotation radius, *R_rc_*, for the Cl-100075 (**top**) and the Ory-107 (**bottom**) anemometers tested each one with all the rotors prepared for this research (see also Table 1).

**Figure 9.**
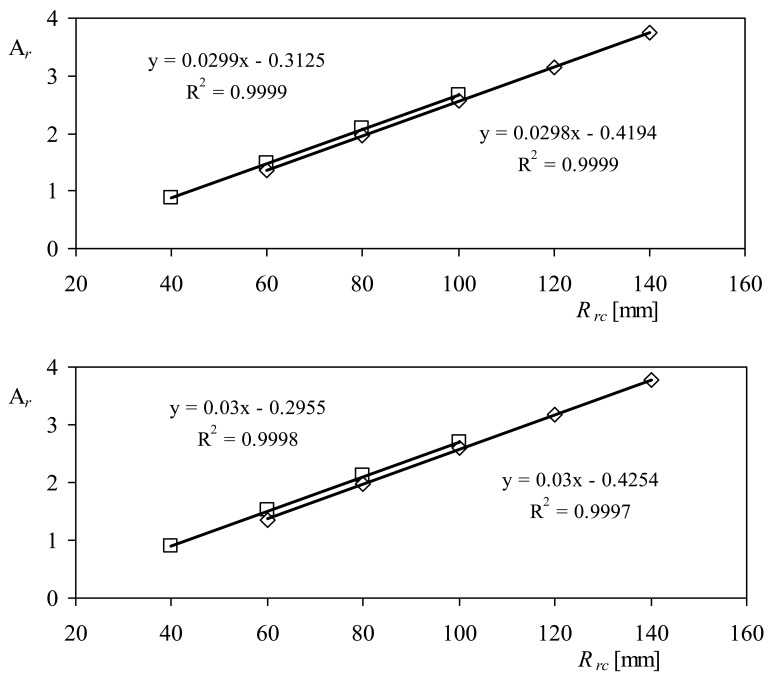
Calibration coefficients, A*_r_*, as a function of the cups' center rotation radius, *R_rc_*, for the Climatronics 100075 (**top**) and the Ornytion 107A (**bottom**) anemometers. Anemometers equipped with 50 mm diameter cups (squares), and 80 mm diameter cups (rhombi).

**Figure 10.**
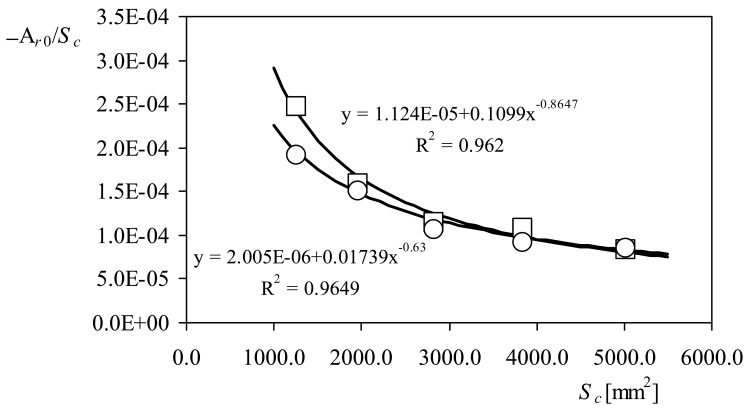
Offset of the linear fittings from Table 2 divided by the cups' front area, A*_r_*_0_/*S_c_*, as a function of the cups' front area, *S_c_*. Data correspond to the Climatronics 100075 (squares) and the Ornytion 107A (circles) anemometers. Power fittings have been also added to the graph.

The calibration results, together with the characteristics of each tested rotor, are included in Table 1. The calibration constants A*_r_*, that is, the anemometers' transfer function slopes, are plotted in Figure 8 as a function of the cups' center rotation radius, *R_rc_*, for the two anemometers tested. A linear fit has been added to both graphs included in the figure. The results seem to be identical for both anemometers, although there is some dispersion due to the different front areas of the cups. In Figure 9, the calibration constants A*_r_* measured in both anemometers equipped with 50 mm and 80 mm diameter cup rotors, are shown as a function of the cups' center rotation radius. The same linear tendency is observed, with better correlation coefficients to the data, *R*^2^. In Table 2, the linear fittings of A*_r_*:

(19) $${\text{A}}_{r}=\frac{d{\text{A}}_{r}}{dR_{rc}}R_{rc}+{\text{A}}_{r0}$$

are included for all cups' diameters. It can be observed that the slopes of all fittings are quite the same, *d*A*_r_*/*dR_rc_* = 0.03 (with *R_rc_* expressed in mm, hereinafter all dimensions concerning cups and rotor geometry will be expressed in mm), whereas the offsets, A*_r_*_0_, are different from one case to another. However, once divided by the cups' front area, *S_c_*, there seems to be a quite good power fitting to these coefficients, when they are plotted against this parameter (see Figure 10). After all these considerations, it has been found that the calibration constant A*_r_* can be expressed as:

(20) $${\text{A}}_{r}=\frac{d{\text{A}}_{r}}{dR_{rc}}R_{rc}-S_{c}\left(\delta +\eta S_{c}^{-\xi }\right)$$

where *δ, η*, and *ξ*, are coefficients that depend on the anemometer (see in Figure 10 the values correspondent to each coefficient for both anemometers tested). The last expression indicates that there is a small contribution to the slope of the anemometers' transfer function that depends on the cups' front area. And this contribution makes the anemometer more efficient in terms of transforming the wind speed into shaft rotation.

The same analysis can be performed on the anemometers' transfer function offset, B. Let's assume that this coefficient has a linear behavior with *R_rc_* as the transfer function slopes:

(21) $$\text{B}=\frac{d\text{B}}{dR_{rc}}R_{rc}+{\text{B}}_{0}$$

**Figure 11.**
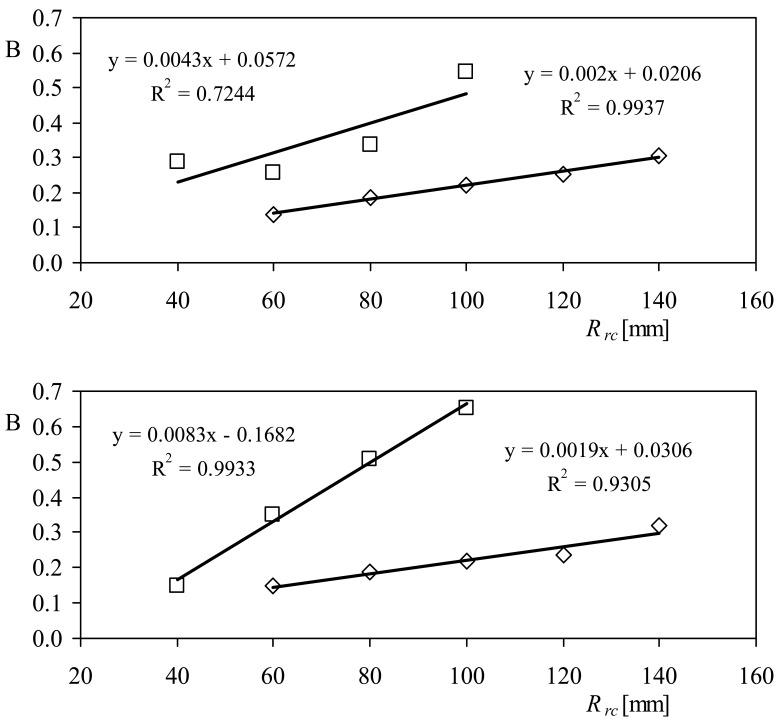
Calibration coefficients, B, as a function of the cups' center rotation radius, *R_rc_*, for the Climatronics 100075 (**top**) and the Ornytion 107A (**bottom**) anemometers. Anemometers equipped with 50 mm diameter cups (squares), and 80 mm diameter cups (rhombi).

**Figure 12.**
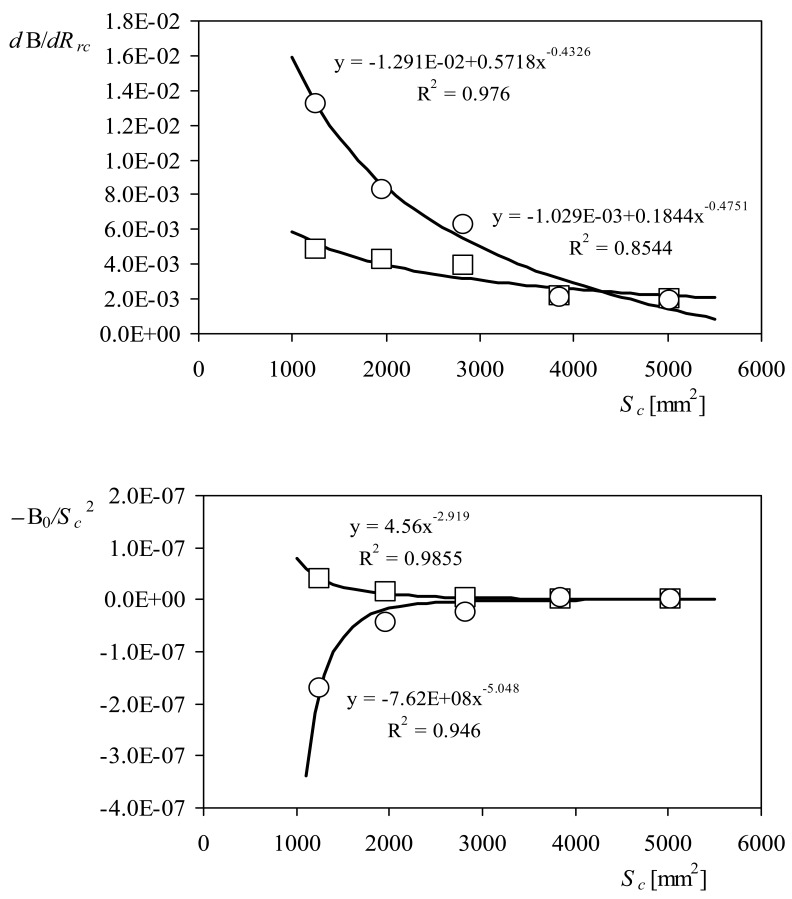
Slope and offset (divided by the squared front area of the cups) of the linear fittings from Table 3, as a function of the cups' front area, *S_c_*. Data correspond to the Climatronics 100075 (squares) and the Ornytion 107A (circles) anemometers. Power fittings have also been added to the graph.

In Figure 11 the behavior of this coefficient is shown as a function of the cups' center rotation radius, *R_rc_*, for the two anemometers equipped with the rotors of 50 mm and 80 mm diameter cups. It can be observed in the figure that the linear fitting is confirmed as quite accurate for describing the coefficient's behavior, despite the fact that some fittings are better than the others (see in Table 3 the linear fittings correspondent to this coefficient). Unlike what happened with the constant, A*_r_*, both the slope, *d*B/*dR_rc_*, and the offset, B_0_, are quite different from one fitting to the other one. Nonetheless, it is possible to find some fitting to describe them as a function of the other important parameter of the rotors' shape, the cups' front area, *S_c_*. In Figure 12, the power fittings to the data concerning the slope and offset of Equation (21) are shown. It can be observed that the power fittings represent the behavior of the aforementioned slope and offset quite well. Based on this fact, Equation (21) can be rewritten as:

(22) $$\text{B}=\left(ɛ+\phi S_{c}^{-\gamma }\right)R_{rc}-\mu S_{c}^{-\psi }$$

where *ɛ, ϕ, γ μ*, and *ψ*, are coefficients that depend on the anemometer (the values correspondent to each coefficient can be derived from the power fittings in Figure 12, for both anemometers tested).

The previous analysis illustrates the relation between both calibration constants, A*_r_* and B, and the two most important shape parameters of the anemometers, the cups' center rotation radius, *R_rc_*, and the cups' front area, *S_c_*. The comparison between the calibration results and the constants estimated with Equations (20) and (22), together with the measured wind speed percentage deviation at *V* = 7 m·s^−1^, is shown in Table 4. The variations with regard to the slope of the transfer function, A*_r_*, are up to 4.9% (Cl-100075) and 2% (Ory-107), whereas with regard to the offset, B, they are larger, up to 43% (Cl-100075) and 211% (Ory-107). These large differences, also between both anemometers, are explained as the offset is mainly produced by the friction [16,17]. It should also be said that the friction term in the expression that describes the anemometer's behavior (3) is affected by changes in the temperature [10]. Therefore, changes in the air temperature during the calibration, together with the different bearings systems of both anemometers can explain the aforementioned differences.

To compare these results with the ones from the classical models it must be taken into account that the anemometer's factor depends on both calibration constants:

(23) $$K=\frac{V}{\omega R_{rc}}=\frac{{\text{A}}_{r}}{2\pi R_{rc}}+\frac{B}{2\pi R_{rc}f_{r}}$$

However, it can be accurate enough to leave aside the second term of this expression, assuming an average deviation up to 8.6% (based on the calibration results in Table 1, the average deviations are 7.4%, 4.1% and 2.8% -Cl-100075-, and 8.6%, 4.7% and 3.2% -Ory-107-, respectively for *V* = 4, 7, and 10 m·s^−1^ wind speed). The results are then *K* = 4.77 and *d*A*_r_*/*dR_rc_* = 0.03, which are a bit far from the results of the 2-cup model (*K* = 3.88 and *d*A*_r_*/*dR_rc_* = 0.0244), Ramachandran model (*K* = 2.64 and *d*A*_r_*/*dR_rc_* = 0.0166), Kondo *et al.* model (*K* = 3.50 and *d*A*_r_*/*dR_rc_* = 0.022), and the previous research [8] (*K* = 3.02 and *d*A*_r_*/*dR_rc_* = 0.019).

**Figure 13.**
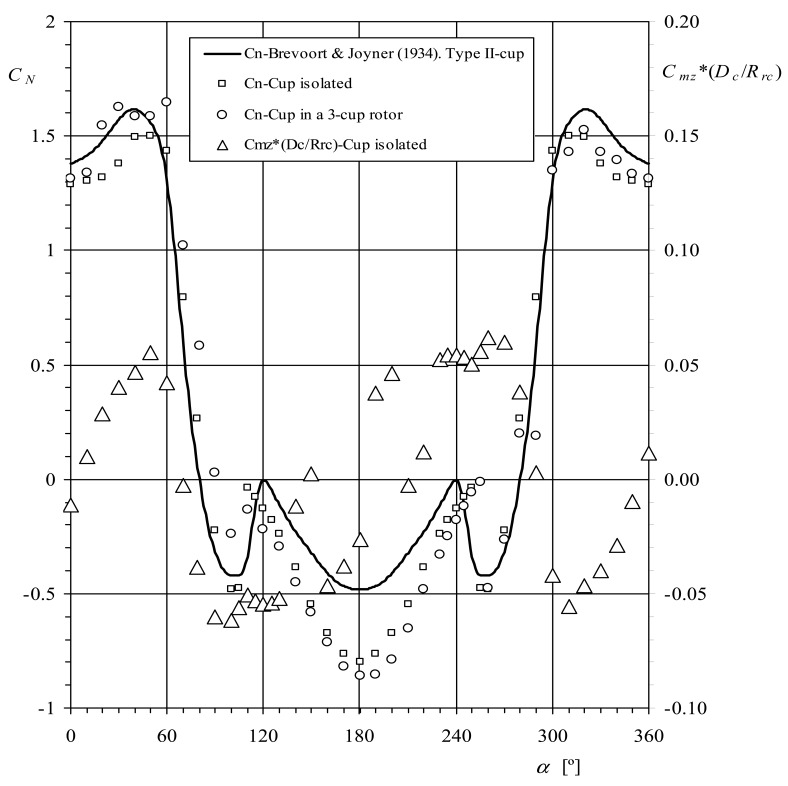
Normal aerodynamic force coefficient, *c_N_*, measured on cup, isolated and surrounded by the other two simulating an anemometer's rotor as a function of the wind direction with respect to the cup, *α*. The aerodynamic yawing moment coefficient, *c_mz_*, multiplied by the ratio *D_c_*/*R_rc_* (*D_c_* = 50 mm and *R_rc_* = 60 mm were selected to calculate this ratio as normal values from commercial anemometers), has been also included in the graph.

The results of the normal aerodynamic force coefficient measured on a cup are included in Figure 13. In this figure the two cases are included, one isolated cup and the measurement cup surrounded by other ones simulating a 3-cup rotor. In this last case, the scaled cup's center rotation radius corresponds to a rotor with *R_rc_* = 60 mm. In the figure, the yawing moment coefficient, *c_mz_*, multiplied by the ratio *D_c_*/*R_rc_* has also been included (see the sketch in Figure 4). As can be observed, the effect of the aerodynamic moment in terms of torque, is much less important than the normal aerodynamic force on the cup. Except for angles between *α* = 120° and *α* = 240°, the experimental results follow the Brevoort & Joyner early results quite well. Nevertheless, a quite large discrepancy is found in that bracket. This can be explained due to the cups' geometries, as the Type II-cup tested by Brevoort and Joyner has rounded edges instead of the sharp edges from the cups tested, and a lower cone-angle (80° instead of 90°).

The use of these experimental results in combination with the described analytical models gives more accurate values of *K* and *d*A*_r_*/*dR_rc_*, when compared to the ones calculated with the Brevoort & Joyner measurements. The results are summarized in Table 5. Notwithstanding, it is possible to go one step further.

**Figure 14.**
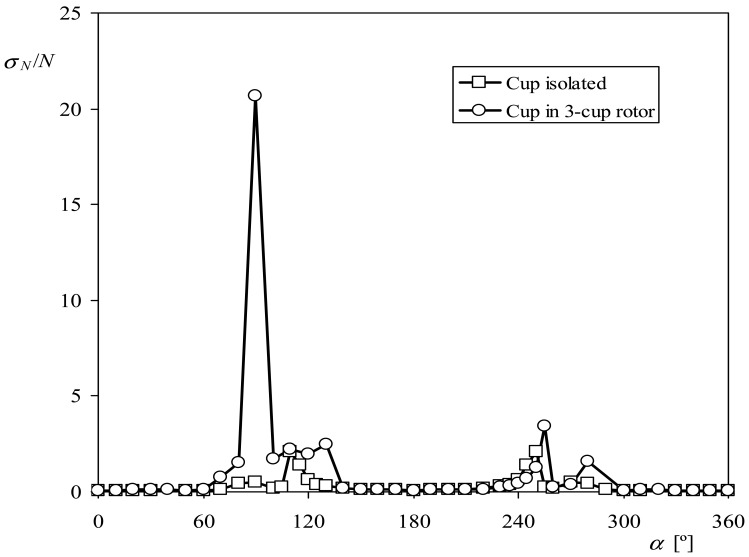
Standard deviation of the normal force on the cup divided by the mean value of this force, *σ_N_*/*N*, as a function of the wind angle with respect to the cup, *α*, in the two cases tested, cup isolated and cup in a 3-cup rotor.

In Figure 14, the standard deviation of the normal force on the cup divided by the mean value of this force, *σ_N_*/*N*, is shown as a function of the wind angle with respect to the cup, for the two cases tested (the isolated cup, and the cup in a 3-cup rotor). In this graph the effect of the wake produced by the upstream cups can be observed. Between *α* = 70° and *α* = 130° the cup in which the forces are measured is clearly in the wake of another, the effect being a dispersion up to 22 times the average force measured. Furthermore, the same effect can be observed between *α* = 255° and *α* = 280°, although in this last case the effect of the non steady aerodynamic effects is less important. It is well known from basic aerodynamics that when a body is in the wake of another, the aerodynamic forces are highly affected. Supposing that in the mentioned intervals the cup is affected by a sudden loss of drag (so the normal force is neglected), the calculations of the anemometer's factor give a quite good result, *K* = 4.75 (*d*A*_r_*/*dR_rc_* = 0.0298), when compared to the result from the calibrations.

## 4. Conclusions

In the present study, the influence of the rotor's shape (cups' front area, *S_c_*, and cups' center rotation radius, *R_rc_*) on the anemometers' performance, has been analyzed by wind tunnel calibrations performed on two different anemometers, tested with 21 different rotors. The results have been compared to those from analytical models improved with data from specific cup aerodynamics wind tunnel testing.

The major conclusions resulting from this work are:

1. Both calibration constants, A*_r_* and B, of a cup anemometer depend on the aforementioned shape parameters. Both constants show a linear relationship with the cups' center rotation radius, *R_rc_*. In the case of constant A*_r_*, the slope of this linear behavior, *d*A*_r_*/*dR_rc_*, only depends on the cups' aerodynamics (and does not depend on any rotor shape parameter), whereas the offset, A*_r_*_0_, seems to depend only on the cups' front area, *S_c_*. In case of constant B, both the slope, *d*B*_r_*/*dR_rc_*, and the offset, B*_r_*_0_, seem to depend only on the cups' front area.
2. The slope of an anemometer's transfer function, that is, the calibration constant A*_r_*, can be accurately estimated with Kondo *et al.* analytical model if it is based on precise aerodynamic data related to the aerodynamics of the rotor cups.

## Figures and Tables

**Table 1.** Results of the calibration performed on the Climatronics 100075 and Ornytion 107A anemometers. The calibration constants, A, B and A*_r_*, are indicated together with the cups' diameter, *D_c_*, and the cups' center rotation radius, *R_rc_*, correspondent to each rotor tested.

**Climatronics 100075**					

**Rotor**	***D****_c_* **[mm]**	***R****_rc_* **[mm]**	**A**	**B**	**A***_r_*

40/40	40	40	0.0310	0.2593	0.9295
40/50	40	50	0.0420	0.3010	1.2592
40/60	40	60	0.0518	0.3562	1.5526
50/40	50	40	0.0293	0.2867	0.8777
50/60	50	60	0.0495	0.2567	1.4850
50/80	50	80	0.0697	0.3387	2.0909
50/100	50	100	0.0890	0.5447	2.6692
60/40	60	40	0.0279	0.1900	0.8361
60/60	60	60	0.0481	0.1559	1.4425
60/80	60	80	0.0682	0.2167	2.0464
60/100	60	100	0.0866	0.3731	2.5991
60/120	60	120	0.1064	0.4731	3.1922
70/60	70	60	0.0461	0.1642	1.3833
70/80	70	80	0.0674	0.1754	2.0210
70/100	70	100	0.0873	0.2003	2.6200
70/120	70	120	0.1067	0.2997	3.1997
80/60	80	60	0.0454	0.1376	1.3633
80/80	80	80	0.0653	0.1864	1.9601
80/100	80	100	0.0859	0.2221	2.5781
80/120	80	120	0.1052	0.2539	3.1557
80/140	80	140	0.1248	0.3040	3.7455

**Ornytion 107A**					

**Rotor**	***D****_c_* **[mm]**	***R****_rc_* **[mm]**	**A**	**B**	**A***_r_*

40/40	40	40	0.4809	0.2739	0.9617
40/50	40	50	0.6396	0.3707	1.2792
40/60	40	60	0.7827	0.5387	1.5654
50/40	50	40	0.4477	0.1479	0.8954
50/60	50	60	0.7584	0.3513	1.5168
50/80	50	80	1.0571	0.5058	2.1141
50/100	50	100	1.3489	0.6509	2.6978
60/40	60	40	0.4313	0.0702	0.8625
60/60	60	60	0.7363	0.1824	1.4727
60/80	60	80	1.0397	0.2957	2.0795
60/100	60	100	1.3143	0.4646	2.6285
60/120	60	120	1.6139	0.5543	3.2278
70/60	70	60	0.7074	0.1848	1.4148
70/80	70	80	1.0275	0.2796	2.0549
70/100	70	100	1.3173	0.2182	2.6347
70/120	70	120	1.6022	0.3444	3.2044
80/60	80	60	0.6794	0.1500	1.3588
80/80	80	80	0.9936	0.1902	1.9873
80/100	80	100	1.3001	0.2174	2.6003
80/120	80	120	1.5906	0.2359	3.1812
80/140	80	140	1.8830	0.3191	3.7659

**Table 2.** Linear fittings (slope, *d*A*_r_*/*dR_rc_*, offset, A*_r_*_0_, and correlation coefficient, *R*^2^) of calibration constants A*_r_* as a function of the cups center rotation radius, *R_rc_*, with regard to calibrations performed on Climatronics 100075 and Ornytion 107A anemometers, equipped with the same rotors. The diameter, *D_c_*, and front area of the rotors' cups, *S_c_*, are also included.

**Climatronics 100075**					

**Rotors tested**	***D****_c_* **[mm]**	***S****_c_* **[mm^2^]**	***d*A***_r_***/*dR****_rc_*	**A***_r_***_0_**	***R****^2^*

40/40, 40/50, 40/60	40	1,256.6	3.116E–02	–3.107E–01	0.99888
50/40, 50/60, 50/80, 50/100	50	1,963.5	2.990E–02	–3.125E–01	0.99986
60/40, 60/60, 60/80, 60/100, 60/120	60	2,827.4	2.934E–02	–3.243E–01	0.99974
70/60, 70/80, 70/100, 70/120	70	3,848.5	3.024E–02	–4.157E–01	0.99953
80/60, 80/80, 80/100, 80/120, 80/140	80	5,026.5	2.980E–02	–4.194E–01	0.99989

**Ornytion 107A**					

**Rotors tested**	***D****_c_* **[mm]**	***R****_rc_* **[mm]**	***d*A***_r_***/*dR****_rc_*	**A***_r_***_0_**	***R****^2^*

40/40, 40/50, 40/60	40	1,256.6	3.019E–02	–2.406E–01	0.99911
50/40, 50/60, 50/80, 50/100	50	1,963.5	3.002E–02	–2.955E–01	0.99980
60/40, 60/60, 60/80, 60/100, 60/120	60	2,827.4	2.943E–02	–3.003E–01	0.99968
70/60, 70/80, 70/100, 70/120	70	3,848.5	2.974E–02	–3.497E–01	0.99923
80/60, 80/80, 80/100, 80/120, 80/140	80	5,026.5	3.004E–02	–4.254E–01	0.99970

**Table 3.** Linear fittings (slope, *d*B/*dR_rc_*, offset, B_0_, and correlation coefficient, *R*^2^) of calibration constants B as a function of the cups center rotation radius, *R_rc_*, with regard to calibrations performed on Climatronics 100075 and Ornytion 107A anemometers, equipped with the same rotors. The diameter, *D_c_*, and front area of the rotors' cups, *S_c_*, are also included.

**Climatronics 100075**					

**Rotors tested**	***D****_c_* **[mm]**	***S****_c_* **[mm^2^]**	***d*B/*dR****_rc_*	**B_0_**	***R****^2^*

40/40, 40/50, 40/60	40	1,256.6	4.8445E–03	6.3273E–02	0.99370
50/40, 50/60, 50/80, 50/100	50	1,963.5	4.2794E–03	5.7171E–02	0.72444
60/40, 60/60, 60/80, 60/100, 60/120	60	2,827.4	3.9178E–03	2.0341E–02	0.83575
70/60, 70/80, 70/100, 70/120	70	3,848.5	2.1567E–03	1.5786E–02	0.81394
80/60, 80/80, 80/100, 80/120, 80/140	80	5,026.5	2.0019E–03	2.0607E–02	0.99374

**Ornytion 107A**					

**Rotors tested**	***D****_c_* **[mm]**	***R****_rc_* **[mm]**	***d*B/*dR****_rc_*	**B_0_**	***R****^2^*

40/40, 40/50, 40/60	40	1,256.6	1.3239E–02	–2.6754E–01	0.97653
50/40, 50/60, 50/80, 50/100	50	1,963.5	8.3167E–03	–1.6821E-01	0.99334
60/40, 60/60, 60/80, 60/100, 60/120	60	2,827.4	6.2523E–03	–1.8674E–01	0.99262
70/60, 70/80, 70/100, 70/120	70	3,848.5	2.0861E–03	6.9016E-02	0.58547
80/60, 80/80, 80/100, 80/120, 80/140	80	5,026.5	1.9197E–03	3.0577E–02	0.93054

**Table 4.** Percentage variations of the anemometer transfer function slope and offset, A*_r_* and B, between the measured values and the estimated ones (based on the geometry parameters, Equations (20) and (22). The measured wind speed variations at *V* = 7 m·s^−1^ wind speed are also included in the table.

**Rotor**	**Climatronics 100075**			**Ornytion 107A**		

	**ΔA***_r_* **[%]**	**ΔB [%]**	**ΔV [%]**	**ΔA***_r_* **[%]**	**ΔB [%]**	**ΔV [%]**

40/40	−3.47%	4.93%	−3.16%	−0.83%	92.54%	2.82%
40/50	−4.92%	7.59%	−4.38%	−1.99%	77.80%	2.24%
40/60	−3.57%	5.49%	−3.11%	−0.75%	46.84%	2.91%
50/40	−0.72%	−29.27%	−1.89%	1.46%	132.63%	4.23%
50/60	−0.92%	10.13%	−0.51%	−0.55%	46.92%	1.83%
50/80	−0.93%	7.08%	−0.55%	−0.27%	36.06%	2.36%
50/100	0.08%	−18.74%	−1.38%	0.40%	32.16%	3.35%
60/40	1.21%	−16.50%	0.73%	0.31%	211.19%	2.43%
60/60	0.25%	42.76%	1.20%	−0.50%	79.73%	1.59%
60/80	−0.01%	32.19%	0.98%	−0.68%	47.77%	1.36%
60/100	1.81%	−6.06%	1.39%	1.40%	17.58%	2.47%
60/120	1.69%	−12.41%	0.74%	1.16%	18.25%	2.51%
70/60	2.72%	9.90%	2.89%	0.61%	2.86%	0.67%
70/80	0.00%	32.78%	0.82%	−1.53%	−9.35%	−1.84%
70/100	0.03%	42.43%	1.25%	−0.43%	45.23%	1.00%
70/120	0.66%	12.71%	1.18%	0.59%	10.40%	1.08%
80/60	2.35%	8.49%	2.47%	1.76%	−43.43%	0.80%
80/80	1.80%	3.59%	1.85%	−0.23%	−40.53%	−1.32%
80/100	0.67%	6.60%	0.86%	−0.67%	−34.94%	−1.74%
80/120	1.26%	10.49%	1.59%	0.05%	−28.06%	−0.90%
80/140	1.33%	6.65%	1.56%	0.45%	−37.96%	−1.30%

**Table 5.** Anemometer factor, *K*, and slope of the A*_r_* calibration constant as a function of the rotation radius, *d*A*_r_*/*dR_rc_*, calculated with the different methods explained in the text. The experimentally measured results are also included in the table.

**Analytical models based on Brevoort & Joyner results** [6,7]		

**Model**	***K***	***d*A***_r_***/*dR****_rc_*

2-cup model [3]	3.88	0.0244
Ramachandran [4]	2.64	0.0166
Kondo *et al.* [5]	3.5	0.022

**Analytical models based on drag force measured on one isolated cup**		

**Model**	***K***	***d*A***_r_***/*dR****_rc_*

2-cup model	8.39	0.0527
Ramachandran	3.37	0.0212
Kondo *et al.*	4.8	0.0302

**Analytical models based on drag force measured on one cup in a 3-cup rotor**		

**Model**	***K***	***d*A***_r_***/*dR****_rc_*

2-cup model	9.51	0.0598
Ramachandran	3.46	0.0218
Kondo *et al.*	4.98	0.0313
Kondo *et al.* with shielding effects	4.75	0.0298

**Experimental results**		

**Testing campaign**	***K***	***d*A***_r_***/*dR****_rc_*

Based on calibrations on commercial anemometers [8]	3.02	0.019
Present research	4.77	0.03
